# Linear interictal pain in Epicrania Fugax

**DOI:** 10.1186/s10194-015-0507-7

**Published:** 2015-03-19

**Authors:** Juan A Pareja, Pablo Bandrés

**Affiliations:** Department of Neurology, University Hospital Fundación Alcorcón, Budapest 1, Madrid, 28922 Alcorcón Spain

## Abstract

Epicrania Fugax is a paroxysmal, short-lasting, head pain moving across one hemicranium, describing a linear or zag trajectory, starting and ending in territories of different nerves. Between attacks, patients are usually free of symptoms. We describe an Epicrania Fugax patient complaining of interictal pain. The interictal pain was line-shaped and extended across the usual starting and ending points of the typical Epicrania Fugax paroxysms. Although rarely encountered, persistent linear pain may be a feature of Epicrania Fugax.

## Correspondence/Findings

*Epicrania Fugax* is a paroxysmal, short-lasting (1–10 s), head pain moving across one *hemicranium*, describing a linear or zag trajectory, starting and ending in territories of different nerves [1,2]. At the end of the attacks, ipsilateral autonomic signs such as lacrimation, conjunctival injection or rhinorrhoea may occur. Between attacks, patients are usually free of symptoms. However, we have recently encountered an *Epicrania Fugax* patient complaining of interictal pain. This finding was not a surprise as interictal pain may be a feature of paroxysmal headaches [3] and trigeminal neuralgia [4].

A 39-year-old female patient complained of one-day-lasting episodes of linear-shaped pain in her right *hemicranium*. In the past few months she had been suffering from paroxysms of severe pain lasting 5–10 seconds, starting in the posterior parietal area of the right *hemicranium* and rapidly moving with a lineal trajectory to the internal *canthus* of the ipsilateral eye. As a rule, once the pain reached the eye, lacrimation ensued. The frequency ranged from 1 to 3 paroxysms daily, in the symptomatic days (2–3/week). In between attacks, the patient felt a continuous, moderate pain, confined to the territory where the motion was perceived. The patient clearly delineated the painful lineal area in her head and could finely draw it, thus giving a definite impression of the localization of such an interictal pain.

Wang Y et al. [5] described headaches with pain episodes localized in a line-shaped area of one *hemicranium*. Topographically, this description parallels that of our patient. However, the patients of Wang Y et al. did not report moving paroxysms. More evidences are needed before we can decipher the nosologic position of linear headaches, but as far as *Epicrania Fugax* is concerned, its clinical features may rarely include a linear interictal pain that may even become the main complaint.
